# On Reproduction of Tendons Whose Divided Ends Have Separated over Four Inches

**Published:** 1862-11

**Authors:** 


### ON REPRODUCTION OF TENDONS

### WHOSE DIVIDED ENDS HAVE SEPARATED OVER FOUR INCHES.

By DR. COOPER.

The curious and very interesting process of reproduction of tendons whose divided extremities have separated several inches,is just beginning to be watched by members of the medical profession with peculiar interest. In times gone by, two inches was fixed upon as the maximum distance in which a separation of the ends of a divided tendon could take place and reproduction occur. Now we regard the matter differently, and boldly cut through contracted tissue, whether it be tendon, ligament or muscle, and restore the deformed part to its proper position, even if the margins of the wound separate five or six inches, having the utmost confidence in the reparation of the proper tissues, whenever the patient's system is in a condition favorable to recovery from any wound whatever.

But in order to secure the reproduction of the same substance to fill the space previously occupied by tendon, it is necessary that the process of granulation be established. Thus, in dividing the tendon of Achilles, if the contraction of the parts were so great as to cause a separation of the divided ends three or four inches, it would not do to resort to the subcutaneous section, and afterwards apply a bandage around the limb, by which the surfaces covering the space occupied by the tendon are pressed together, because an early union would take place, and thereby prevent the reproduction of tendon. It is necessary, in that case, to cut down through the skin to as great an extent as the ends of the divided tendon are disposed to separate, and, after the operation, fill the space thus made with lint, so that it must heal throughout by granulation. This is the only method upon which perfect reliance can be placed for the reproduction of tendinous substance in the place where it has been removed to any considerable extent. By this method, the old rule of never permitting the ends of a divided tendon to separate from each other more than two inches, becomes, as it should be, obsolete.

When this is the case, and surgeons, throughout the world, no longer regard the admission of atmosphere into the tissues of the extremities as a sort of danger, orthopœdic surgery will make advances never before known or dreamed of.

The question will be, in the progress of surgery, when operating for deformities of the hands or feet—What blood-vessels and nerves, likely to be involved in the operation upon the tissues, must be divided to a sufficient extent to admit of an easy removal of the deformity?

To be more definite, surgeons, when operating for talipes varus, in cases where there is extensive true fibrous degeneration, involving a great amount of tissue, will not hesitate to cut down into the anterior part of the sole of the foot, when the plantar arterial branches have become small, and divide the entire tissues, if requisite, to the bony structure, even if it should become necessary to invade some of the metatarso-phalangial joints, or of the tarso-metatarsal articulations. In the latter operation, it may become necessary, in some instances, to ligate the internal plantar artery, but, if so, it will be no particular impediment to the success of the operation.

Our mode of operating for clubfoot is, to divide a sufficient amount of contracted tissue to insure a removal of the deformity, let that be great or small. We have frequently cut down through the sole of the foot, directly to the bony structure, in cases of clubfoot, and invariably with the best of results. Under ten years old, no case of clubfoot is incurable, if the surgeon's practice be guided by the above principles.

We wish this statement to be taken as absolute, because no case of clubfoot, in early life, is beyond the reach of surgery. Of course, we do not wish to be understood to say, that a feeble state of health may not prevent success in these cases, because that may be a barrier to the success of any treatment, and in any kind of case. But we mean to say, that, ordinarily, cases of clubfoot are curable, without the least regard to the extent of deformity, and though we occasionally failed, in former times, in consequence of being guided, more or less, by the old rules of surgical writers, we have never done so on this coast, and do not expect to do so, unless unavoidably interrupted in the process of treatment.

Many persons may be inclined to the opinion that this extensive section of contracted tissue is an unnecessarily severe operation. To this we answer, *first*, that success and safety will vindicate any operation, and, *second*, that it is a very mild method, compared with the torturing plan of curing the deformity by the application of tightly fitting apparatus. The latter keeps the patient in pain for weeks, or even months, whereas the former only for a few moments, when the painful period is over forever.—*San Francisco Medical Press.*

